# Applicability of hyperspectral imaging during salinity stress in rice for tracking Na^+^ and K^+^ levels *in planta*

**DOI:** 10.1371/journal.pone.0270931

**Published:** 2022-07-07

**Authors:** Isaiah Catalino M. Pabuayon, Irish Lorraine B. Pabuayon, Rakesh Kumar Singh, Glen L. Ritchie, Benildo G. de los Reyes

**Affiliations:** 1 Department of Plant and Soil Sciences, Texas Tech University, Lubbock, Texas, United States of America; 2 International Rice Research Institute, Los Baños, Laguna, Philippines; ICAR - National Research Center on Plant Biotechnology, INDIA

## Abstract

The ratio of Na^+^ and K^+^ is an important determinant of the magnitude of Na^+^ toxicity and osmotic stress in plant cells. Traditional analytical approaches involve destructive tissue sampling and chemical analysis, where real-time observation of spatio-temporal experiments across genetic or breeding populations is unrealistic. Such an approach can also be very inaccurate and prone to erroneous biological interpretation. Analysis by Hyperspectral Imaging (HSI) is an emerging non-destructive alternative for tracking plant nutrient status in a time-course with higher accuracy and reduced cost for chemical analysis. In this study, the feasibility and predictive power of HSI-based approach for spatio-temporal tracking of Na^+^ and K^+^ levels in tissue samples was explored using a panel recombinant inbred line (RIL) of rice (*Oryza sativa* L.; salt-sensitive IR29 x salt-tolerant Pokkali) with differential activities of the Na^+^ exclusion mechanism conferred by the *SalTol* QTL. In this panel of RILs the spectrum of salinity tolerance was represented by FL499 (super-sensitive), FL454 (sensitive), FL478 (tolerant), and FL510 (super-tolerant). Whole-plant image processing pipeline was optimized to generate HSI spectra during salinity stress at EC = 9 dS m^-1^. Spectral data was used to create models for Na^+^ and K^+^ prediction by partial least squares regression (PLSR). Three datasets, *i*.*e*., mean image pixel spectra, smoothened version of mean image pixel spectra, and wavelength bands, with wide differences in intensity between control and salinity facilitated the prediction models with high R^2^. The smoothened and filtered datasets showed significant improvements over the mean image pixel dataset. However, model prediction was not fully consistent with the empirical data. While the outcome of modeling-based prediction showed a great potential for improving the throughput capacity for salinity stress phenotyping, additional technical refinements including tissue-specific measurements is necessary to maximize the accuracy of prediction models.

## Introduction

High-throughput and high-resolution physiological phenotyping has become a much greater limitation (more than genotyping) in characterizing large populations for genetic studies as well as individual plant selection in breeding. Accurate scaling of quantitative variation is critical for understanding the interaction between the genotype and the environment (GxE) in phenotypic expression [1]. While physiological profiling of a smaller subset of individuals in a population present only minor challenges and poses virtually no limitation in terms of resolution, dealing with much larger populations that are often required for trait dissection by QTL mapping or Genome-wide Association Studies (GWAS) tend to be more time-consuming, resource-intensive, and subject to inaccuracies and inconsistencies [2].

Physiological phenotyping for salinity tolerance is a critical step in dissecting the genetic basis of such quantitative and polygenic trait. For instance, differential uptake of Na^+^ by plant cells through non-selective K^+^ channels as measured by Na^+^/K^+^ ratio is an important measure of salinity stress injuries as it reflects the extent of cellular toxicity [3]. Variation in Na^+^/K^+^ ratio between individuals across a genetic population is evaluated by examining the differences in tissue ion content by destructive sampling, and further by direct chemical analysis in the laboratory [4–6]. While it is a standard procedure, chemical analysis of a large number of individuals across a genetic population tends to be labor intensive, costly, and prone to inaccuracies and inconsistencies that could undermine the biological significance of the data. Furthermore, destructive tissue sampling does not allow a continuous observation of physiological and morphological changes in a spatio-temporal scale when treatment effects should be monitored during the course of plant growth and development.

Imaging-based approaches for non-destructive physio-morphometric profiling has recently offered a more robust and high-throughput alternative for assessing quantitative differences across a genetic population. These approaches allow profiling of individuals in real-time, permitting simultaneous or parallel investigation of multiple physiological, biochemical, and morphological parameters in every single individual across the population in a single experiment [7]. For example, numerous studies have employed a high-throughput phenotyping strategy for simultaneous screening of the effects of salinity, drought, and disease-causing pathogens within the same genetic populations of rice (*Oryza sativa* L.) and upland cotton (*Gossypium hirsutum* L.) [8–12]. These studies facilitated the identification of quantitative trait loci (QTL) for tolerance, which was made possible by the high digital resolution and quantitativeness of the overall physiological and developmental state of each individual plant in the segregating or recombinant population [13].

Accumulation of Na^+^ and K^+^ in plant tissues under salinity stress is typically determined by chemical analysis through flame photometry [4]. As this approach involves destructive tissue sampling, simultaneous measurements of other parameters on the same individual plant becomes a major limitation that confounds the correlation of other traits with Na^+^/K^+^, which can only be addressed with increased number of individual plant replicates for every single genotype in the data matrix. Furthermore, destructive sampling of tissues for chemical analysis creates discontinuous observations as the same individual plant in a population could not be observed throughout developmental stages for robust biological interpretation of spatio-temporal changes due to stress effects.

In this study, we evaluated the potential of Hyperspectral Image (HSI) analysis as a non-invasive approach for quantifying the Na^+^ and K^+^ accumulated in plant tissues during salinity stress using a comparative panel of recombinant inbred lines (RILs) of rice representing a gradient of salinity tolerance, derived from a cross between the salt-sensitive *indica* cultivar IR29 and salt-tolerant *aus* cultivar Pokkali [14]. The principle of HSI is governed by characteristic signatures of macromolecular or elemental absorption of radiation in genetically, developmentally, and/or physiologically distinct individuals, which is then used as an index to determine the quantity, spatial distribution, and temporal fluxes of the absorbing macromolecule or element within an individual plant [15,16]. HSI has also been applied for evaluating overall plant health, growth and nutritional status in large population studies through the ‘*normalized difference vegetation index*’ (NDVI) or ‘*photochemical reflectance index*’ (PRI) [17]. The wide range of spectral wavelengths used in HSI analysis makes it possible to capture different aspects of plant physiological and biochemical properties through the integration of wavelengths from visible (400–700 nm), near-infrared (NIR; 750–1400 nm), and short wavelength infrared (SWIR; 1400–3000 nm) [18].

HSI has been used to discriminate foliar biochemical status between plant species, and to evaluate the effects of abiotic and biotic stresses on plant health and vigor. The most common example is the measurement of nitrogen and chlorophyll contents as indices of overall plant health and vigor [19–23]. More recently, HSI-based physiological profiling has been applied to investigate the levels of elemental macronutrients and micronutrients in a number of crop species [24,25]. In this study, we applied the same principles of HSI to establish models for predicting Na^+^ and K^+^ levels in rice plants subjected to salinity stress using three different spectral datasets created by different methods of data normalization. Using the IR29 x Pokkali RIL comparative panel, we evaluated the physiological and biological significance of the predictive models and their potential applications as proxy to direct chemical analysis in large population studies. We uncovered the refinements that are necessary for a robust application of HSI-based modeling for the estimation and prediction of Na^+^ and K^+^ levels for future applications in large-population studies in plant genetics and breeding.

## Methods

### Experimental design and hyperspectral imaging

RILs representing different levels of salinity tolerance were selected from the IR29 X Pokkali F_8_ population. As previously described, this minimal comparative panel included the parents IR29 (salt-susceptible) and Pokkali (salt-tolerant), and the transgressive segregants FL510 (super-tolerant), and FL499 (super-sensitive), and two individuals representing the parental phenotypic range, FL478 (tolerant) and FL454 (susceptible) [14]. Briefly, to establish the plants for HSI experiments, dehulled seeds were first disinfected with ethanol (70% v/v) and bleach solution (50% v/v) before germination in 0.5X Murashige-Skoog (MS) agar for 4 to 5 days. Seedlings (n = 10 for each genotype) were subsequently established in hydroponics using the Turface MVP® as solid media in Yoshida nutrient solution [26]. Five plants each (n = 5) were used for the control and salinity stress experiments. Treatment plants were exposed to elevated levels of salt after establishment for 14-days and observed through the Scanalyzer 3D imaging platform (LemnaTec, GmbH, Aachen, Germany) at the facility of the University of Nebraska-Lincoln for a total of 19 days. Salinity stress treatment was administered by adding NaCl:CaCl_2_ solution (270 mM NaCl:9.9 mM CaCl_2_), which increased the EC (electrical conductivity) of the nutrient solution to 4.5 dS m^-1^ (~45 mM NaCl). This initial salinity treatment was escalated the next day to EC = 9 dS m^-1^ (~90 mM NaCl) and maintained at that level through the duration of the experiment [14,27].

The HSI datasets used in modeling were from previously published phenomics study [14,28] (Dryad/*doi*:*10.5061/dryad.2jm63xsrm*) where observations were generated through an established protocol that employed an extended VNIR (visible and NIR) push-broom type imaging spectrometer camera (Headwall Photonics, Fitchburg, MA, USA) [24]. In this dataset, images were taken from the side view of the plant, with a spectral range of 550 to 1700 nm (green-red region to proximal part of SWIR), and spectral interval of 4.77 nm for individual images. In total, each plant had 243 pictures corresponding to the spectral range for each day throughout the duration of the salinity stress experiments. Plants were imaged daily during the afternoon (1400 H to 1600 H) for a total of eighteen (18) times for a period of nineteen (19) days, with the exception of day-7, for which no images were collected due to unforeseen technical issues in the imaging system. This observation period encompasses the V4 (early tillering) to V12 (maximum tillering) stages of rice development [14].

### Image capture and processing, ion content modeling, and statistical analysis

Image processing included three steps–masking, cropping, and pixel counting. Masking removed the non-plant pixels from the images acquired by HSI. Mask was created for each image group folder using images captured from the red-orange band (~615 nm) and the middle of the NIR region (~1117 nm) that was selected arbitrarily to create NDVI images. These images were used to eliminate the majority of non-plant pixels that could provide noises on the data.

To capture the exact plant area, images were further cropped and cleaned through the removal of non-plant pixels (*i*.*e*., bucket, stand, background) from the raw image datasets. Mean pixel intensities were counted first through the pixel intensities for every pixel used to establish the means, such that even the background pixels with zero (0) intensities were counted. Secondly, mean pixel intensities were also counted by using only the non-zero pixels to minimize the contribution of background pixels to the overall mean. The first approach had issues with plant sizes affecting the intensity, since the number of background pixels with zero intensity lowered the mean, while the second method did not.

The pixel intensities of each image folder were assembled to create the reflective spectrum for each plant on a given day. Using the *prospectr* package, the reflective spectra were modified in some of the analyses by smoothening the curves to remove background noises [29]. The spectra were smoothened via gap-derivative smoothing approach, which combined the gap-segment filtering and the derivatization of the spectra to reduce noise and smoothen the curves, which was an important aspect of enhancing the data consistency for prediction.

Ion content models were created using the partial least squares regression (PLSR) of the image spectra from the last day of the experiment (18 days after salinity stress or DAS), and empirical Na^+^ and K^+^ content measurements made on the same samples via flame photometry (described in the next section). Samples from the last day of imaging were used for empirical quantification of Na^+^ and K^+^ contents to limit the damage being made to the plants during the imaging period. Mechanical damage may elicit different responses that may be picked up by HSI, leading to erroneous spectra being created. Samples taken on the last day of the experiment were also theoretically the most divergent between the two treatments, as a result of longest duration of exposure to salinity stress. Thus, the correlations made between the spectra and the ion contents quantified through flame photometry were expected to be highly contrasting between the treatments.

Being a commonly used method for establishing predictive models in chemometrics studies, the PLS regression analysis was used in this study for a number of reasons [30]. First, the strength of this method lies in its capacity to analyze large datasets with inherent noises or those with highly collinear variables, such as absorbance spectra. Second, in comparison to other regression methods such as stepwise regression analysis or the use of normalized difference vegetation indices (NDVI), PLSR utilizes more data-points rather than discarding majority of the data available, making its output more robust [31].

The *pls* package in ‘R’ was used in the regression modeling [32]. A total of forty-eight (48) samples were used for training the model. To maximize the accuracy of the model, a total of twenty-four (24) samples (*i*.*e*., half of the total dataset) were used to construct a PLSR model, which was tested against the remaining samples (*i*.*e*., the other half of the total dataset). At the same time, the number of components that gave the best prediction were determined by the root mean squared error of prediction (RMSEP). The coefficient of linear regression (R^2^) value was used to assess the accuracy of prediction. This process was repeated 100,000-times to get the model with the lowest RMSEP, highest R^2^ value, and the number of components (out of 10) that created these parameters. The model was applied to the rest of the spectral dataset to establish estimates of the daily ion content in each genotype in the comparative RIL panel. Graphical representations of analysis outputs were created using the ‘*ggplot*’ package [33].

### Chemical analysis of ion content by flame photometry

Independent experiment identical and parallel to the experiment performed for HSI data collection was performed to collect samples for chemical analysis of Na^+^ accumulation by flame photometry. Plants of the exact same RILs used for HSI experiments were grown for 14 days in nutrient solution, after which they were exposed to salinity stress by adding NaCl:CaCl_2_ solution (270 mM NaCl:9.9 mM CaCl_2_) to increase the EC of the nutrient solution to 4.5 dS m^-1^ (~45 mM NaCl). Like in the HSI experiments, salinity was further elevated after 24 hours to EC = 9 dS m^-1^ (~90 mM NaCl) and maintained at that level for 18 days. Control experiment without salt amendment was performed concurrently under the same conditions in the greenhouse. Shoot samples were collected 6, 12, and 18 days after salinity stress exposure to establish the temporal profile of ion accumulation, with three (3) biological replicates per genotype and for each treatment at each sampling date.

The established protocol of Munns et al. [4] was slightly modified and used as reference for this procedure. Shoot tissues (100 mg) were pulverized and weighed into 15 mL tubes, and then suspended in 10 mL of 0.5 M nitric acid (HNO_3_). Mixture was gently shaken in an oven at 37°C for 72 hours, and then centrifugated at 4000 rpm for 20 minutes. This step was necessary to separate the plant debris from the solution and avoid clogging the flame photometer. The supernatant was diluted to 2% concentration (*i*.*e*., 0.1 mL of sample + 4.9 mL of deionized H_2_O) to reduce the ion content of the solution within the detection ranges of the flame photometer below saturation, and thus within the linear level. A standard curve (40 ppm, 30 ppm, 20 ppm, 10 ppm, 5 ppm, and 1 ppm) was created from 1000 ppm Na^+^ and K^+^ standard solutions for quantifying the ion contents of the samples, and as a quality check for the procedure.

## Results

### Filtered and optimized datasets

**Fig 1:**
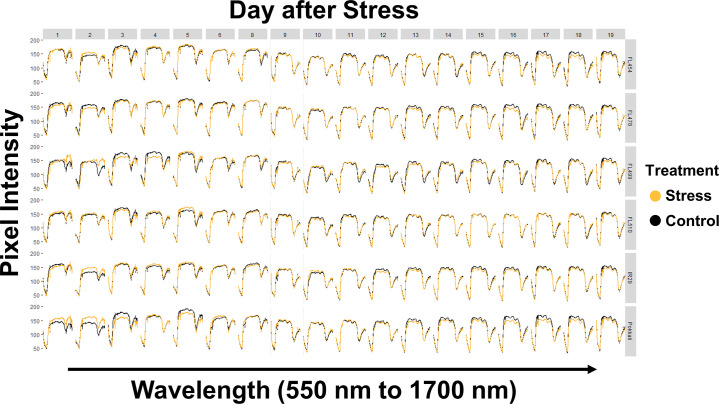
Spectral graphs using mean pixel intensities according to genotype and days after salinity treatment. Average pixel intensity for each image at a given wavelength was used to create spectral graphs for each day (x-axis) and for each genotype (y-axis). Non-zero pixel intensities were used to prevent bias due to higher number of pixels with zero (0) value during the earlier stages of plant growth.

**Fig 2:**
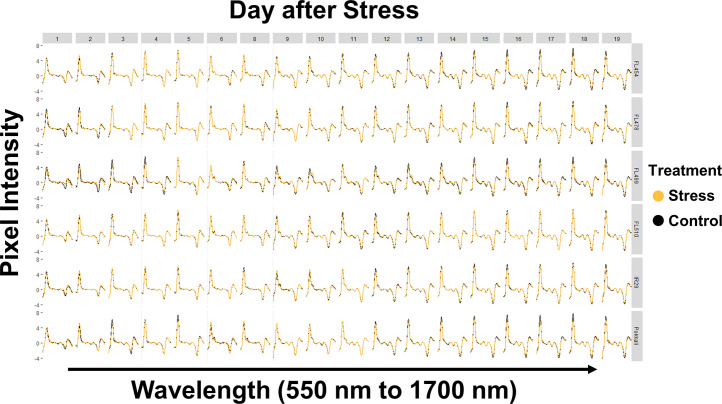
Spectral graphs of the data cleaned by smoothening through gap-segment derivatization, presented by genotype across different durations of salinity stress. Spectral data used to create **Fig 1** was cleaned and smoothened using a moving average (gap-segment) and 1^st^ degree derivatization, which emphasized the peaks. This approach also removed the random changes in pixel intensity, which lead to smoother curves that emphasized real differences between treatments.

**Fig 3:**
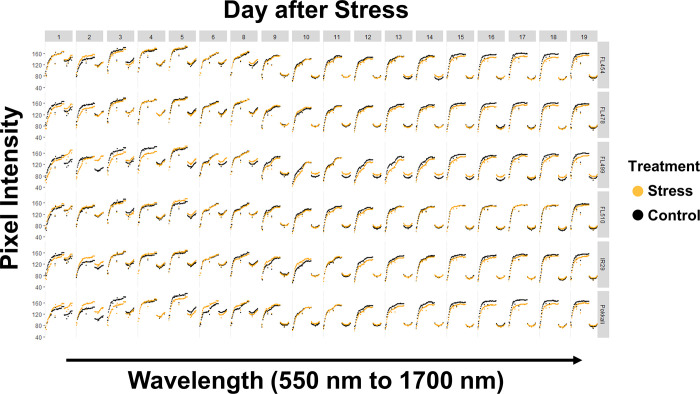
Spectral graphs based on wavelengths showing high magnitude of differences between treatments, presented by genotype across different durations of salinity stress. This dataset was generated by calculating the quartiles of pixel intensities in each wavelength, after which only the wavelengths that have a difference of more than the 3^rd^ quartile between stress and control were selected. This approach intended to remove the wavelengths that may be non-contributory for the creation of PLSR models, which can sufficiently distinguish between salinity and control. However, this method also removed a substantial amount of data, which appeared to undermine the predictive power of the model.

The comparative genotypic panel used in this study was comprised of the salt-sensitive *indica* cultivar IR29 (parent), salt-tolerant and *SalTol*-donor *aus* landrace Pokkali (parent), and four of their derived recombinant inbred lines (F_8_-RILs) representing the spectrum of salinity tolerance within the parental range (*i*.*e*., FL454 = *SalTol*-negative, and salt-sensitive like IR29; FL478 = *SalTol*-positive and salt-tolerant like Pokkali), and outside the parental range (*i*.*e*., FL499 = *SalTol*-negative and more sensitive than IR29 and FL454 hence *super-sensitive*; FL510 = *SalTol*-positive but more tolerant than Pokkali and FL478 hence *super-tolerant*) [14]. Two identical subsets of plants representing the genotypic comparative panel were grown in parallel under normal (control) condition, and salinity stress condition that progressed from EC = 4.5 dS/m for one (1) day to EC = 9 dS/m for another 17 days. Hyperspectral images of each individual in the parallel control and salinity stress experiments were captured at a bandwidth range of 550 nm to 1700 nm (green-red region to proximal part of SWIR). The daily spectral traces for each plant based on mean pixel intensity described in previously published work [14] are shown in **Fig 1**. Data showed that the spectra contain many inconsistencies, which could adversely affect the accuracy of regression models for prediction. Two possible solutions to this issue were tested, first by smoothening the spectral data with a gap-segment derivatization method **(Fig 2)**, and second by selection of specific wavelengths with the highest differences in pixel intensity **(Fig 3)**. The smoothening process removed small crests and troughs in the spectra and emphasized the wavelength segments with high differences in intensity [29]. On the other hand, selection of specific wavelengths with the highest differences in pixel intensity was more straightforward. Wavelengths with a difference between control and salinity that are higher than the third quartile of the entire pixel intensity dataset were used. This approach appeared to have improved the regression model by removing potentially uninformative wavelengths. However, both approaches also appeared to have the drawback of removing data-points that are highly likely to be *bona fide* observations that can be legitimately used in modeling. The smoothened dataset reduced the number of data-points per plant from 243 to 223 (8.23% reduction), and the quartile-derived set included only 61 of the total wavelengths (75% reduction) for analysis.

### Correlation between hyperspectral and wet-lab measurements

**Fig 4:**
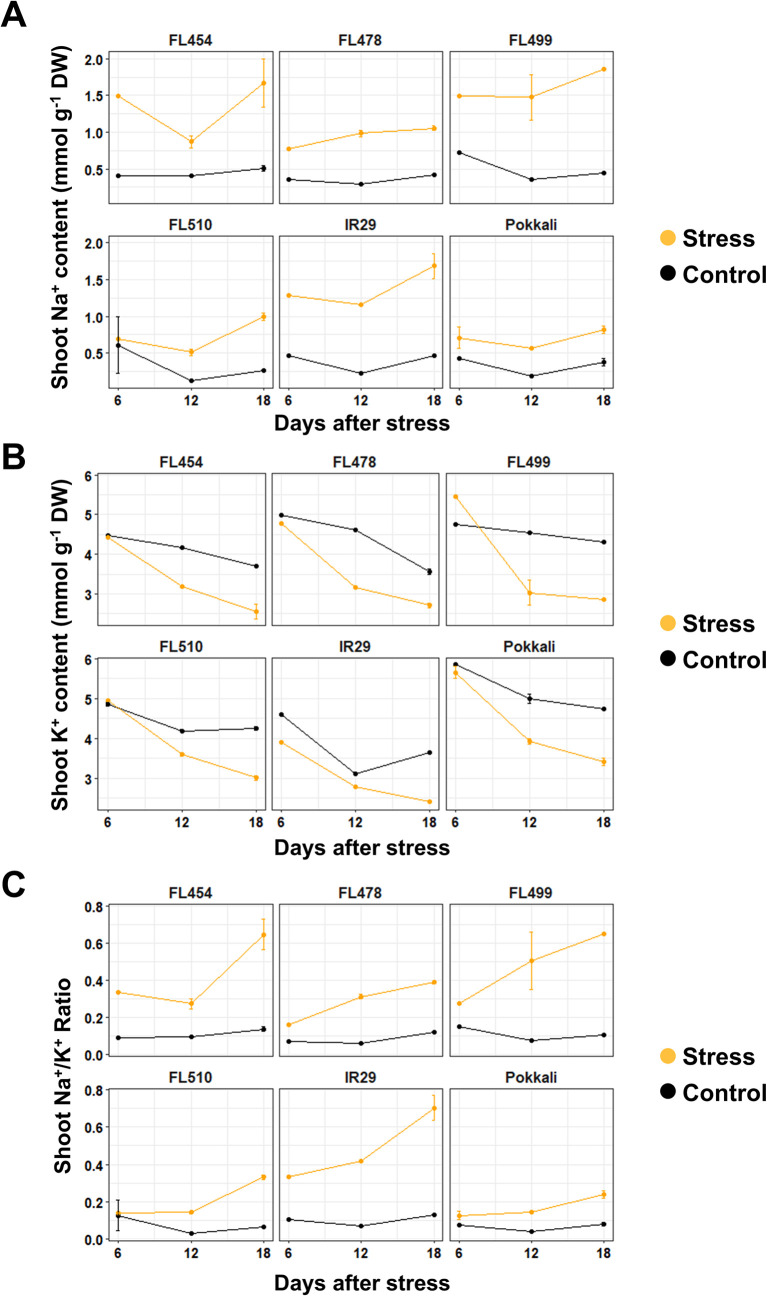
Empirical measurements of tissue Na^+^ and K^+^ contents across different durations of salinity stress as measured by flame photometry analysis. Plants were sampled at three evenly spaced time-points (6, 12, and 18 DAS). Na^+^ content was stable under control condition but markedly elevated under salinity stress. Tolerant genotypes (*i*.*e*., donor parent Pokkali, FL510, FL478) had lower Na^+^ content compared to susceptible genotypes (*i*.*e*., recipient parent IR29, FL454, FL499) throughout the duration of the experiment. This trend implies that the difference in Na^+^ content started before the earliest sampling time-point (6 DAS). All genotypes showed a decreasing trend in K^+^ accumulation. There was little difference in K^+^ content in first sampling time-point, which increased with exposure time. These trends were similar across genotypes, implying that in terms of K^+^ accumulation, different genotypes appeared to have the same response regardless of their inherent tolerance or sensitivity to salinity.

To test the validity of the trends in ion accumulation that was revealed by HSI, parallel control and salinity experiments identical to the set-up used for image capture was performed for chemical analysis of tissue Na^+^ and K^+^ contents 6, 12, and 18 days after salinity stress (DAS; **Fig 4A and 4B)**. While Na^+^ levels were elevated relative to K^+^ in all genotypes under salinity stress, Na^+^/K^+^ ratio was significantly higher among the salt-sensitive genotypes (recipient parent IR29, FL454, FL499) than the salt-tolerant genotypes (donor parent Pokkali, FL478, FL510; **Fig 4C**). Changes in Na^+^ levels under stress started as early as 6 DAS, especially in the sensitive genotypes **(Fig 4A)**. In contrast, in all genotypes, decline in K^+^ levels started at 12 DAS (**Fig 4B)**. Increase in Na^+^ concentration coincided with the onset of growth stagnation, which was observed most prominently in the sensitive genotypes [14]. In contrast, in the tolerant genotypes, decline in growth rate was attenuated until 2 to 3 days after the sensitive genotypes had shown drastic declines in growth rate. In the sensitive genotypes, difference in growth between control and salinity were much larger compared to the tolerant genotypes. This indicates that the accumulation of Na^+^ had a very significant effect on the plant’s health in the sensitive genotypes, while the tolerant lines had other mechanisms that appeared to be effective in mitigating the toxicity of elevated salt.

Partial Least Squares Regression (PLSR) models (n = 48) were constructed on the ion concentration measurements and corresponding HSI data during the last day of imaging. During the last day of imaging, individual plants were destructively sampled without the compromising the integrity and quality of images since image capture was already completed. The total dataset was divided randomly into two subsets (50%-50% split), and utilized as ‘*training dataset’* and ‘*test dataset*’. By iterative and repeated resampling of the *training dataset*, the best fit PLSR models were constructed until the best regression coefficient (R^2^) for the *test dataset* with its best component number was determined. In testing the model to predict Na^+^ and K^+^ levels, the R^2^ values obtained for the PLSR projections were at a reasonable range.

**Fig 5:**
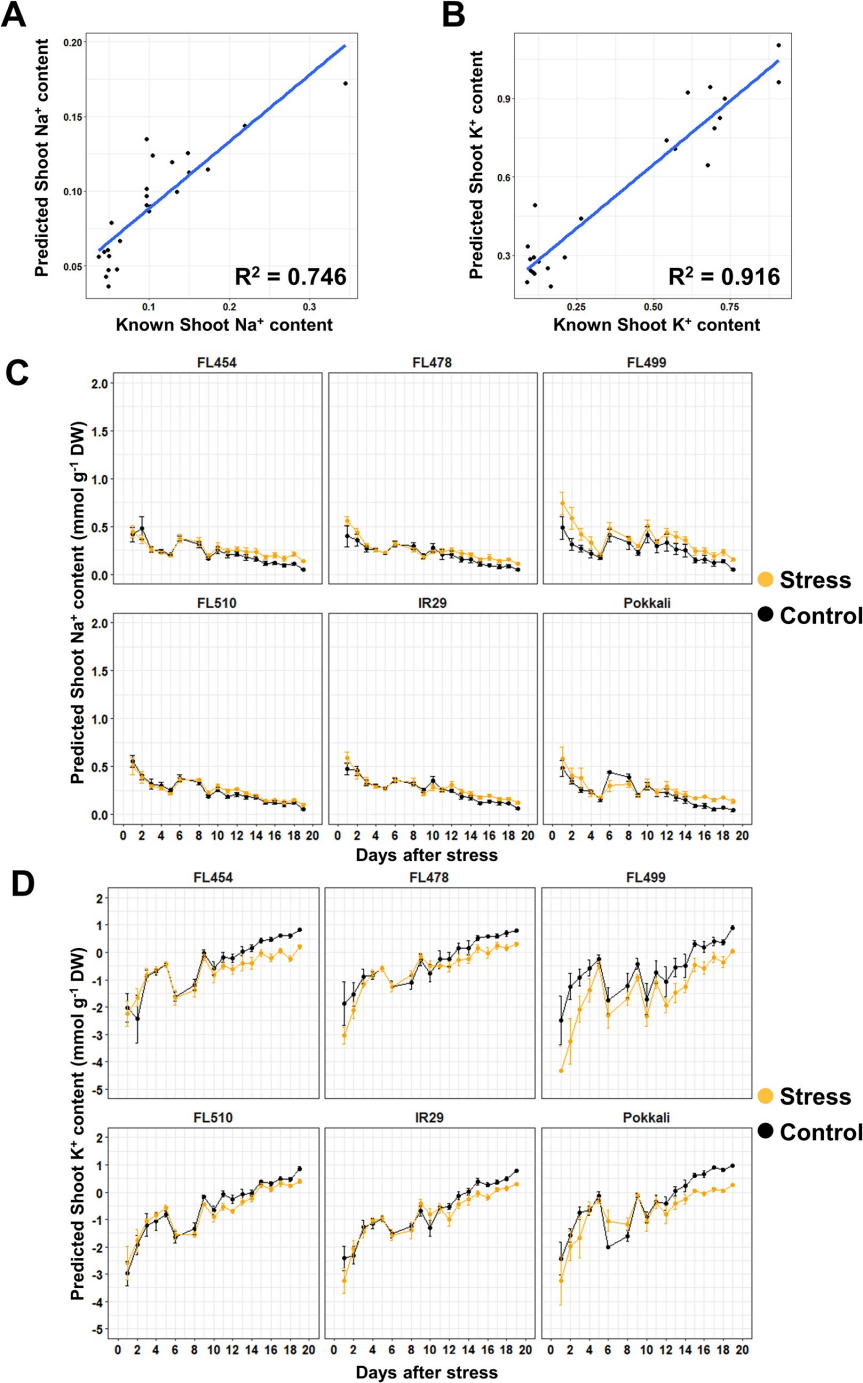
Trends in ion accumulation predicted by PLSR and the corresponding linear regression of the PLSR model created from the mean image pixel intensity. The accuracy of the model was tested by creating a scatterplot between the samples not included in training the PLSR model, and their corresponding Na^+^ (**A**) and K^+^ (**B**) content predictions. While the models for both ions had satisfactory R^2^ values, the data-points tend to cluster in two groups. The model was further used to predict ion accumulation trends for Na^+^ (**C**) and K^+^ (**D**). Images taken during the last day of imaging were used in conjunction with the empirical ion content values that were determined by flame photometry. Overall, the trends in Na^+^ and K^+^ accumulation were opposite. However, the trends are also opposite from what is expected based on flame photometry. Despite this result, there is a definite deviation between salinity and control for both Na^+^ and K^+^.

The initial dataset on mean pixel intensity spectra **(Fig 1)** had R^2^ values of 0.746 and 0.916, and CV (*i*.*e*., cross-validation estimate of the number of components for the best model) values of 0.056 and 0.292 for Na^+^ and K^+^, respectively **(Fig 5A and 5B)**. However, the trends established by PLSR were not totally in agreement with the empirical measurements. Overall, Na^+^ contents in all genotypes showed a downward trend regardless of treatment **(Fig 5C)**. Slight deviations between treatments started between 9 DAS to 12 DAS in all genotypes except in the super-tolerant FL510. The super-sensitive FL499 had the largest deviation between treatments. The trends in K^+^ accumulation were essentially opposite of the trends in Na^+^ accumulation **(Fig 5D)**. Deviation between treatments were also observed in the same time period. However, the accuracy for K^+^ accumulation is questionable due to the negative values predicted for earlier time-points. It is possible that such deviations may have been due to the clustering of samples used in regression **(Fig 5A and 5B)**. In addition to the inaccuracies in the regression model, the range of values predicted for this dataset were much lower than what was observed in the empirical measurements for both Na^+^ and K^+^ **(Fig 4)**.

**Fig 6:**
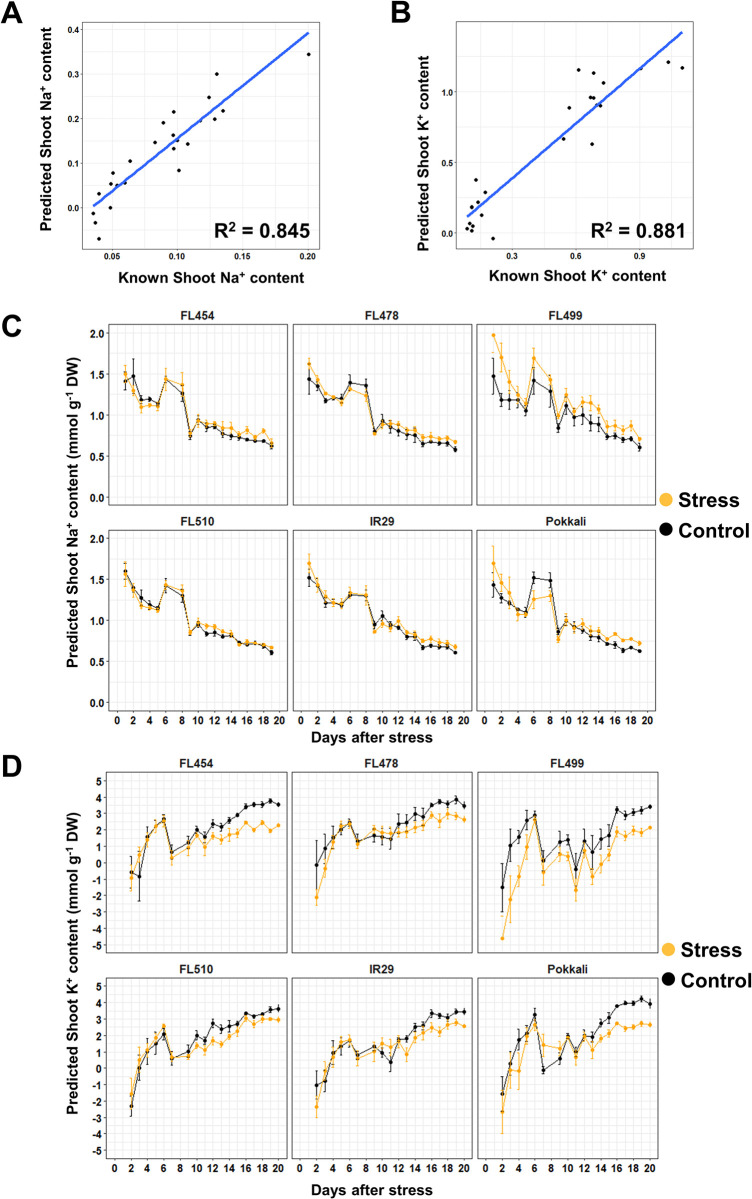
Trends in ion accumulation predicted by PLSR and the corresponding linear regression for the dataset cleaned by gap-segment derivatization. The initial dataset used for creating the ion accumulation model in **Fig 2** was smoothened and derivatized to the first degree. This approach removed the inconsistent peaks in the spectra, creating a more consistent data distribution. The regression scatterplots for testing the model accuracy are shown for Na^+^ (**A**) and K^+^ (**B**). For this dataset, the spread of data-points in the regression plot for Na^+^ was improved compared to the previous dataset, while the regression plot for K^+^ had the same clustering as previously observed. The predicted ion accumulation trends for Na^+^ (**C**) and K^+^ (**D**) are shown. As with **Fig 2**, the patterns of ion accumulation predicted by the PLSR model showed an opposite trend to what was expected based on flame photometry data. The scales for the prediction graphs had also changed compared with the initial dataset, as the predicted values were higher in general for this dataset.

Similar trends were observed for the other two datasets. For the smoothened dataset **(Fig 2)**, R^2^ for Na^+^ and K^+^ were 0.845 and 0.881, with CV of 0.056 and 0.291, respectively **(Fig 6A and 6B)**. In general, the predicted values for Na^+^ increased especially during the earlier time-points **(Fig 6C)**. This was also true for the predicted K^+^ values, however, negative values were still predicted **(Fig 6D)**. Deviation between treatments were also smaller in this dataset compared to the original dataset, perhaps because of the higher values in general. The regression line for Na^+^ accumulation using this dataset was more evenly spread across the trendline compared to the original dataset, which gives a better validation to the regression **(Fig 6A)**. However, the regression line for K^+^ also clustered into two main groups, which appeared to be causing the prediction of negative values **(Fig 6B)**. Despite this, the range of values predicted in this dataset matched better with the range of values determined experimentally (**Fig 4).** This is probably due to the reduction of noise by smoothening, and greater emphasis of treatment differences by derivatization. The similarity in the range of values increased the accuracy of the model, making it viable for ion content prediction.

**Fig 7:**
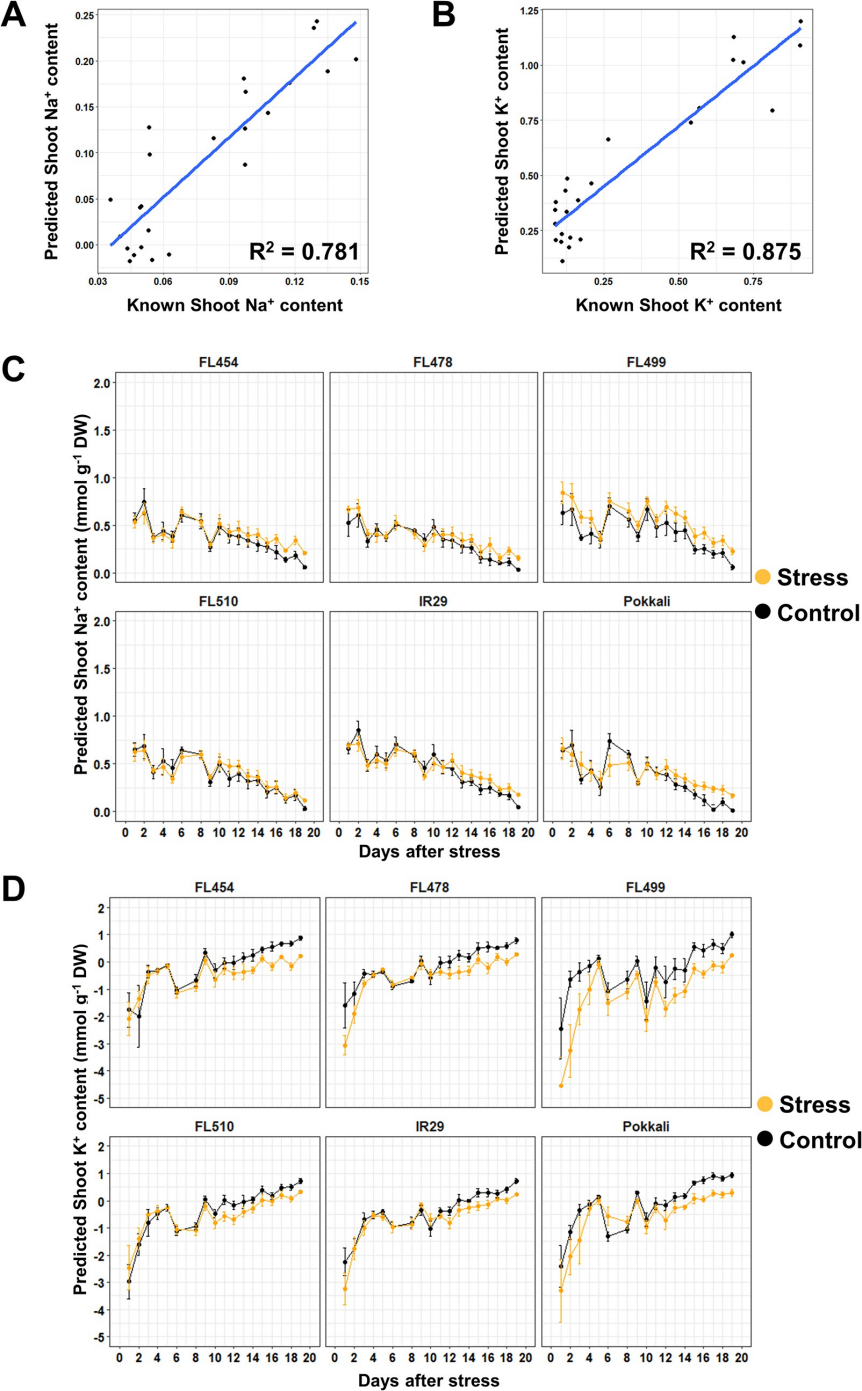
Trends in ion accumulation predicted by PLSR and the corresponding linear regression graphs for the dataset that included only the wavelengths with high mean difference between control and salinity. The dataset used in **Fig 2** was filtered for wavelengths with substantial mean difference between control and salinity. Difference in the means of control and salinity across genotypes were calculated and the third quartile values were used as threshold for selecting the wavelengths. In total, 67 out of 243 bands were used for the PLSR model. As the wavelengths no longer form a continuous selection of points like the other datasets in **Figs 2** and 3, no additional smoothening and derivatization was necessary. The regression scatterplot for Na^+^ (**A**) also showed an improved spread compared to the plots in **Fig 2**. However, its R^2^ value was lower compared to that in Fig 3. The regression scatterplot for K^+^ (**B**) showed clustering of points into two groups in spite of high R^2^ value. Trends in ion accumulation for Na^+^ (**C**) and K^+^ (**D**) are shown, which appeared to be more similar to those shown in **Fig 2**.

The third dataset filtered out 61 wavelengths with wider differences between control and salinity across genotypes **(Fig 3)**. For Na^+^ and K^+^, the R^2^ were 0.781 and 0.875, with CV of 0.066 and 0.294, respectively **(Fig 7A and 7B)**. The trends for both ions remained mostly the same with the other two datasets **(Fig 7C and 7D)**. The scale of values was also more similar to the first dataset for both ions. The points in the regression line of Na^+^ were also more evenly spread out, like in the second dataset **(Fig 7C).** However, for K^+^, there was also an observed clustering into two groups, which is consistent with the other datasets **(Fig 7D).** Similar to the first model **(Figs 1** and **5)**, this model appeared to underestimate the values of Na^+^ and K^+^ based on the total range. This indicates that while the uninformative wavelengths may have been removed in this dataset leading to better R^2^ values, the informative wavelengths basically gave the same prediction as what was seen in the initial dataset. This result shows that noise reduction and emphasis on differences through derivatization is more important than just removing uninformative data points. While the inaccuracy of the predicted values makes this dataset less desirable for further analysis, it also shows consistency with the first data set. This indicates that the process for creating models could be transferrable to other experiments but would require pristine datasets to make accurate and usable prediction outputs.

## Discussion

The models created by PLSR yielded high R^2^ values that gave confidence to the trends revealed despite having some minor inconsistencies. The values for K^+^ in the early time-points were especially concerning, as they indicate negative concentrations. There is also an apparent difference with the trends revealed from the flame photometry data and those predicted by HSI. Several factors may have contributed to these observations. One of them is the assumption that the pixel measurements for whole plant are uniform throughout (*i*.*e*., from one organ to another or from one tissue to another). However, experiments have shown that this may not be the case, and different cells, tissues, or organs could have different ion concentrations due to both cell-to-cell and long distance transport mechanisms [34,35]. Thus, the tissues used for flame photometry as well as the specific area used for image analysis and construction of spectra need to be reassessed and synchronized in order to reduce the total variance and improve accuracy. The image capture process may also present variabilities that reduce the consistency of data, thus careful maintenance of the image capture process is necessary.

The smoothened and derivatized dataset had the best R^2^ value (0.845) for Na^+^ concentration. The low values for K^+^ in this dataset were also higher and closer to zero than the others **(Fig 6D)**. In addition, the R^2^ value for K^+^ concentration did not deviate much from the other datasets, and it had the best spread of data-points of all the datasets **(Fig 6B).** This may be due to the removal of noise that interferes with modeling. Differences are also emphasized through the derivatization of the values. In comparison, filtering out wavelengths as applied to the third dataset may be an improvement over the raw spectral data, but this may also result in the loss of informative data-points. The tighter clustering of points in the K^+^ regression models for the unprocessed spectra **(Fig 4B)** and filtered spectra **(Fig 7B)** may also have created inaccurate models with high regression values. The tight cluster tend to reduce residual values and increase R^2^, but less data-points at higher values means lower accuracy in predicting ion concentrations. Thus, the more evenly distributed data-points in the smoothened and derivatized dataset **(Fig 6B)** can be assumed to be more accurate than the selected dataset. The accuracy of this dataset is further supported by the similarity in the range of predicted values to the empirical dataset (**Fig 4).** In contrast, the other two datasets vastly underestimated the ion contents of the sample by around 50% (*i*.*e*., up to 1.0 mmol g^-1^ DW for the empirical set, up to 0.5 mmol g^-1^ DW for the predicted set), making their applications in actual experiments unrealistic.

The analysis may also necessitate the sampling and quantification of samples in the middle of the temporal observation period (*i*.*e*., seven days after stress) to establish the relationship between spectral data and ion quantities without having large disparities between stress and control. K^+^ contents of plants under salinity vary greatly compared to normal conditions [36,37]. This may also be contributory to the congregation of data points in the linear regression models for K^+^. In the case of the rice genotypes used in this study, the main difference would seem to be in their Na^+^ accumulation patterns **(Fig 4A)**, as all of them showed steady downward accumulation of K^+^ under salinity **(Fig 4B)**. This is in line with the previous observation that Pokkali confers strong potential for salt exclusion to its tolerant progenies FL510 and FL478, possibly through effects of the *SalTol* QTL [14,38].

The trends established by PLSR indicate that there may not be a wide difference between the ion contents of plants under normal and saline conditions at the whole plant level. For Na^+^, the overall concentration lowered through time. It was noticeable that the super-tolerant FL510 had very similar values in the control and salinity experiments. Meanwhile, the others had only small deviations, especially FL499, which was the most sensitive to salinity. Thus, while differences are small at the whole plant level, it may be possible to differentiate genotypes based on the deviation of Na^+^ concentration, especially during extended stress periods. The trends shown by K^+^ accumulation also indicate that there is a definite maintenance of K^+^ content in the more superior genotypes like FL510. K^+^ content has been observed to decrease with increasing magnitude of salt stress [39,40]. This may be contributory to the decrease in growth as metabolic processes such as photosynthesis are perturbed [41,42]. Thus, maintenance of K^+^ or supplementation of K^+^ helps alleviate these symptoms [37,43].

Small differences across genotypes also point to how small alterations in the concentration of Na^+^ and K^+^ at the whole plant level could severely hamper growth. However, this may also indicate that there are other factors aside from ion accumulation that create osmotic and toxicity effects under salinity. Thus, it may be necessary to look at other indices in conjunction with ion accumulation to fully classify phenotypic variation. Specifically, spatial Na^+^ accumulation patterns may be the critical determinant of tolerance. Similar findings were found by Ahmadizadeh et al. [44], which showed how salt accumulates in older tissues first and such spatial dynamics have been positively correlated with the magnitude of tolerance. More thorough image analysis and ion quantitation will enable the study of spatial salt accumulation dynamics. This will necessitate increasing the number of images and angles (*i*.*e*., spatial resolution) taken to create more detailed accumulation profiles of different areas within the plant.

This study presents a possible non-destructive approach for salinity tolerance phenotyping. While there are technical issues need to be resolved to further fine-tune and enhance the accuracy of prediction, the current results showed promise in distinguishing highly tolerant genotypes from those that are susceptible (*i*.*e*., binary classification). Thus, this method can be used to minimize flame photometry and destructive chemical analysis, substantially reducing costs, and time for screening. It presents a viable option to scale-up phenotyping, which is a primary bottleneck in accelerating the process of breeding crops for stress tolerance.

## Conclusion

Hyperspectral imaging is rapidly emerging as a viable alternative to destructive chemical analysis of plant tissue samples in assessing plant injuries due to nutrient, osmotic, and ion toxicity stresses. Its primary advantage of being able to perform quantitative tests on the same set of samples from a population at a reduced cost makes it an extremely attractive method for plant selection in genetic studies and plant breeding. It can also potentially offer ways to establish continuous and real-time nutrient accumulation trends in intact plants. In this study, high R^2^ values were achieved between empirically derived ion quantities and HSI data. Both data filtration methods helped improve the model quality, with smoothening and derivatization leading to better accuracy. However, technical issues such as inconsistencies between empirical data and the model still remain. These issues could potentially be improved by increasing the resolution of the images, focusing on specific plant areas (organs or tissues), and using such spatial specificity for empirical measurements and also for reducing other potential sources of variability in the images. The analysis method should also be applied independently to additional genetic populations that have been validated for segregation for salinity tolerance, in order to validate its accuracy. In the current study, differences between sensitive and tolerant genotypes were detected in terms of Na^+^ and K^+^ accumulation, especially for the transgressive RILs FL499 (super-sensitive) and FL510 (super-tolerant). However, trends that are established differ from what was initially hypothesized, especially since Na^+^ trends lowered and K^+^ continuously increased under both treatments. Overall, HSI has the potential to significantly reduce the labor and cost needed for phenotyping of genetic populations for salinity tolerance. The accuracy of this method will certainly depend on additional refinements that requires spatially targeted imaging of specific organs that act as sinks of excess ions. The need for spatially targeted approach to imaging is justified by the dynamic nature of Na^+^ and K^+^ ions *in planta* as dictated by cell-to-cell and long distance transport mechanisms.
